# Plasma C‐terminal agrin fragment concentrations across adulthood: Reference values and associations with skeletal muscle health

**DOI:** 10.1002/jcsm.13507

**Published:** 2024-06-07

**Authors:** Jedd Pratt, Evgeniia Motanova, Ludmilla Pessanha, Marco Narici, Colin Boreham, Giuseppe De Vito

**Affiliations:** ^1^ Department of Sport and Exercise Sciences Manchester Metropolitan University Institute of Sport Manchester UK; ^2^ Institute for Sport and Health University College Dublin Dublin Ireland; ^3^ Department of Biomedical Sciences, CIR‐Myo Myology Centre, Neuromuscular Physiology Laboratory University of Padova Padua Italy; ^4^ Conway Institute of Biomolecular and Biomedical Research University College Dublin Dublin Ireland

**Keywords:** Biomarkers, C‐terminal agrin fragment, Normative data, Sarcopenia, Screening

## Abstract

**Background:**

Increasing interest surrounds the utility of blood‐based biomarkers for diagnosing sarcopenia. C‐terminal agrin fragment (CAF), a marker of neuromuscular junction stability, is amongst the most promising candidates; however, a dearth of reference data impedes the incorporation of its use in public health settings. This study aimed to establish reference values for plasma CAF concentrations across adulthood in a large, well‐characterized cohort of healthy adults; and comprehensively examine the association between plasma CAF levels and skeletal muscle health.

**Methods:**

One thousand people aged between 18 and 87 years took part in this study (mean age = 50.4 years; 51% females). Body composition and muscle strength were examined using DXA and hand dynamometry. Plasma CAF concentrations were measured, in duplicate, using commercially available ELISA kits. Sarcopenia and individual sarcopenia signatures [low skeletal muscle index (SMI) only/low grip strength only] were classified using the EWGSOP2 algorithm.

**Results:**

Detailed reference CAF values, according to sex and age, are presented. A significant but modest age‐related increase in plasma CAF concentration was observed (*P* = 0.018). Across adulthood, CAF concentrations were negatively associated with grip strength and SMI (both *P* < 0.001). In people ≥50 years old, CAF concentrations were 22.6% higher in those with sarcopenia (*P* < 0.001), 11.3% higher in those with low SMI (*P* = 0.006) and 9.6% higher in those with low grip strength (*P* = 0.0034), compared with controls. People in the highest CAF concentration quartile, had 3.25 greater odds for sarcopenia (95% CI = 1.41–7.49, *P* = 0.005), 2.76 greater odds for low SMI (95% CI = 1.24–5.22, *P* = 0.012), and 2.56 greater odds for low grip strength (95% CI = 1.07–5.57, *P* = 0.037), compared with those in the lowest quartile. People with a CAF *Z*‐score ≥2 had 9.52 greater odds for sarcopenia (95% CI = 3.01–30.05, *P* < 0.001) compared with a *Z*‐score <1. Plasma CAF concentration had an acceptable level of diagnostic accuracy for sarcopenia (AUC = 0.772, 95% CI = 0.733–0.807, *P* < 0.001).

**Conclusions:**

The reference values presented herein may guide the clinical interpretation of circulating CAF and help identify people at risk of poor skeletal muscle outcomes for inclusion in therapeutic interventions. Our findings add clarity to existing data, demonstrating a robust relationship between circulating CAF and skeletal muscle integrity in the largest adult cohort to date, and support the use of CAF as an accessible, cost‐effective screening tool for sarcopenia. However, further research into the prognostic utility of plasma CAF, and the establishment of normative data from other populations, are urgently needed if routine CAF screening is to be embedded into public healthcare settings.

## Introduction

Identifying scalable strategies for screening skeletal muscle health across adulthood is critical for the effective management of sarcopenia, a disease of ageing that progressively manifests itself as a decline in muscle mass and function.^1^, ^2^ Sarcopenia affects over 40% of those aged >80 years^3^, ^4^ and imposes a heavy burden on patients and economies globally, due to its association with adverse health outcomes,^5^, ^6^ and correspondingly substantial healthcare costs.^7^ These outcomes will become more prevalent with a progressively ageing global population, underscoring the urgency of identifying effective preventative and management strategies.

Although sarcopenia typically affects those in older adulthood, the decline in skeletal muscle health begins earlier, from 45 to 50 years of age.^8^ Accordingly, screening for and promotion of skeletal muscle health throughout the lifespan are critical for maintaining the integrity of skeletal muscle that underpins functionality in older age. Despite advances in research over recent decades, there remains an urgent need to develop feasible screening strategies that can be employed throughout adulthood and facilitate timely engagement with pre‐emptive measures, such as exercise.

In this regard, blood‐based screening has gained credence as a strategy with potential scalability, which is supported by accumulating evidence demonstrating the indicative relevance of circulating biomarkers to skeletal muscle health.^9^, ^10^, ^11^, ^12^ In particular, C‐terminal agrin fragment (CAF), a marker of neuromuscular junction stability, is amongst the most promising candidates.^13^, ^14^, ^15^ However, although there is increasing evidence to support the potential of blood CAF concentrations as an indicator of skeletal muscle decline, a lack of high‐quality reference data impedes the clinical translation of its use. Moreover, despite the growing recognition that circulating CAF screening may represent an accessible means of identifying people suffering from, or at risk of poor skeletal muscle health,^10^, ^13^, ^14^, ^15^, ^16^ the absence of reference values means that this encouraging approach cannot be acted upon in public health settings. In this regard, establishing normative values would help facilitate an incorporation of CAF screening into routine medical practice, where the translation of existing research may begin to be realized. Furthermore, the data generated in this study may help to stimulate further research into the clinical utility of circulating CAF, and the establishment of normative values for other populations that are essential if a widespread incorporation of CAF assessment is to occur.

There also remains a need to clarify some inconsistencies in existing research relating to the strength of the relationship between circulating CAF concentrations and skeletal muscle traits. Indeed, while existing research largely favours a strong connection between circulating CAF and skeletal muscle health, there are some conflicting findings that require further investigation (summarized in Monti et al.^10^). These inconsistencies may be driven by small sample sizes and lack of adjustment for important covariates, such as habitual physical activity and presence of co‐morbidities, which are likely to mediate the association between circulating CAF and skeletal muscle status.^10^

Accordingly, the primary aim of this study was to establish reference values for plasma CAF concentrations across adulthood in a large (*n* = 1000), well‐characterized sample of healthy adults aged between 18 and 87 years. The secondary aim was to examine the association between circulating CAF concentrations and skeletal muscle health, with a particular focus on sarcopenia signatures.

## Methods

### Study sample

Participants were recruited from the GenoFit study, a large, cross‐sectional analysis of the Irish population examining the relationship between genetics, health, and lifestyle.^17^ Between September 2017 and October 2020, 10 546 individuals attended a single assessment during which blood samples and a range of health and lifestyle data were collected. The sample for the present study was refined to include 1000 randomly selected people aged between 18 and 87 years. Inclusion criteria included being free from severe cognitive and neurological disorders absence of renal function abnormalities (determined via self‐reported medical questionnaire; details in Covariates section and list of specific conditions in the Supporting Information), and being able and willing to provide written informed consent. Ethical approval was granted by University College Dublin's Research Ethics Committee, and written informed consent was obtained from all participants.

### Body composition and muscle strength analysis

Dual energy X‐ray absorptiometry (DXA; Lunar Prodigy, GE Healthcare Technologies, Chicago, IL) was used to measure appendicular lean mass. Skeletal muscle index (SMI) was calculated as the combined lean mass of the limbs divided by height squared (kg/m^2^). Hand grip strength was determined using a digital hand‐held dynamometer (Jamar, JLW Instruments, Chicago, IL) according to a previously described protocol.^8^ In brief, in a standing position with their arm positioned straight by their side, each participant performed two maximal efforts with each hand (3 s duration). The average of the maximal attained value for each hand was considered in the analysis.^18^ Sarcopenia and individual sarcopenia signatures (low SMI only and low grip strength only) were classified according to the EWGSOP2 definition and recent cut‐points proposed by Westbury et al.^4^, ^19^ Sarcopenia was diagnosed upon the presence of low SMI and low grip strength. Corresponding thresholds for low SMI were <7 kg/m^2^ for males and <5.5 kg/m^2^ for females and for low grip strength were <35.5 kg for males and <20 kg for females.

### Blood sampling and plasma C‐terminal agrin fragment measurement

Blood samples were drawn from the median cubital vein using ethylenediaminetetraacetic acid (BD Vacutainer®) collection tubes. Blood was spun at 4°C at 4000 g for 10 min, after which the plasma was pipetted into cryofreeze tubes and stored at −80°C until analysis. Plasma concentrations of CAF were quantified, in duplicate, by enzyme‐linked immunosorbent assay (#ab216945, Abcam, Cambridge, UK) as previously described.^13^ Briefly, 50 μL of diluted sample (six‐fold dilution) or standard and 50 μL of the CAF antibody were added to the pre‐coated wells and incubated at room temperature for 1 h on a plate shaker at 400 rpm. Wells were then washed three times, and 100 μL of TMB development solution was added to each well and incubated for 8 min in the dark on a plate shaker at 400 rpm. Finally, 100 μL of stop solution was added to each well, the microplate was shaken for 1 min to mix, and the measurements were recorded at 450 nm (Infinite F50 Plus, Tecan, Männedorf, Switzerland). Concentrations of CAF were determined by interpolation of a standard curve and corrected for dilution factor. The inter‐assay coefficient of variation was below 9%, and the intra‐assay coefficient of variation was below 5%.

### Covariates

Habitual physical activity, educational attainment, presence of diseases/disorders, alcohol consumption, smoking status, and medication use were determined using previously employed self‐reported questionnaires.^20^ The specific questions were: ‘How many days per week do you do at least 30 min of moderate‐intensity exercise that increases your breathing and heart rate (e.g. brisk walking, jogging, cycling, swimming)?’; ‘What is the highest level of education you have completed to date (no formal education, primary, lower secondary, higher secondary, third level, or postgraduate)?’; ‘Have you ever received a medical diagnosis from a doctor for any of the following conditions?’ (full list in Supporting Information); ‘On average, how many standard drinks do you drink per week?’; ‘Smoking categorized as (1) never smoked (never smoked/smoked <100 cigarettes in lifetime), (2) previous smoker (smoked ≥100 cigarettes in lifetime but no longer smoking), and (3) current smoker (smoked ≥100 cigarettes in lifetime and currently smoking)’; ‘Please list any medications you are currently taking (including antibiotics, oral/implant contraceptive or hormone replacement therapy)’.

### Statistical analyses

Data are presented as mean ± standard deviation (SD), unless otherwise stated. Residual plots and skewness and kurtosis data were inspected to assess normality. Percentile curves for plasma CAF concentrations across adulthood were developed using the lambda‐mu‐sigma (LMS) method.^21^ Independent‐sample Student's *t* test and chi‐square tests examined differences in subject characteristics according to sex, and Pearson's correlation and multiple linear regression models assessed the association between plasma CAF, age, and skeletal muscle health in the full sample. Analyses of variance assessed the differences in plasma CAF, grip strength, and SMI according to disease/disorder prevalence. In people aged ≥50 years, analyses of covariance examined the association between plasma CAF concentrations and sarcopenia status (healthy, low SMI only, low grip strength only, or sarcopenic). We chose ≥50 years of age as the cut‐off as our group have previously observed that a noticeable decline in skeletal muscle health is observable from this point.^8^ To establish the clinical value of the normative data presented herein, we examined the odds for sarcopenia and individual sarcopenia signatures according to plasma CAF concentration quartile and *Z*‐score. *Z*‐scores were defined as the difference between the individual CAF value and the mean CAF value from a young reference population (18–39 year olds from the GenoFit cohort), divided by the SD of the young reference population (young reference mean = 2569.1 pg/mL and SD = 535.5 pg/mL). For *Z*‐score analysis, we grouped people into the following categories: <1, 1–1.99 and ≥2; and focussed on sarcopenia with a minimal model of adjustment (sex, age, and BMI), in consideration of the small sample sizes in the 1–1.99 and ≥2 *Z*‐score groups (*n* = 44 and *n* = 20). Receiver operating characteristic analyses were performed to determine the diagnostic utility of plasma CAF for sarcopenia and individual sarcopenia components, with optimal cut‐points established by Youden's index. Thresholds for interpreting AUC values were 0.5–0.69 = poor, 0.7–0.79 acceptable, 0.8–0.89 = good, and 0.9–1.0 excellent. Models were adjusted, when relevant, for a panel of confounders including age, BMI, sex, habitual physical activity, co‐morbidity, smoking status, educational attainment, and alcohol consumption. Statistical analyses were conducted using SPSS (Version 28, IBM SPSS Inc., Chicago, IL, USA), and MedCalc (Version 22.016, MedCalc Software, Ostend, Belgium), with *P* < 0.05 as the threshold for statistical significance threshold. Data visualizations were developed using GraphPad Prism (Version 9.3.1, Prism, San Diego, CA, USA).

## Results

Detailed participant characteristics according to sex are presented in Table 1. A total of 1000 adults aged between 18 and 87 participated in this study (males: *n* = 494, mean age = 50.2 years, age range = 18–87 years; females: *n* = 506, mean age = 50.6 years, age range = 18–87 years). Compared with females, males had a lower disease prevalence (*P* < 0.001), more frequently engaged in smoking (*P* = 0.014), consumed more alcohol (<0.001), and were more physically active (*P* = 0.039). Although no statistically significant differences were observed, plasma CAF concentrations appeared to be positively related to disease/disorder prevalence, with differences of up to 25% being observed between those with ≥2 diseases/disorders compared with those with none (≥80‐year age category; *Table* *S1*). In contrast, grip strength and SMI tended to be lower in people with a higher prevalence of diseases/disorders, with differences of up to 22% and 12%, respectively (≥80‐year age category; *Table* *S1*). A total of 221 participants were medicating (males: *n* = 85; females, *n* = 136), for which the principal reasons were contraception (*n* = 30), hypertension (*n* = 27), asthma (*n* = 22), hypercholesterolemia (*n* = 17), and hypothyroidism (*n* = 16).

**Table 1:** Participant characteristics

Parameter	Total (*n* = 1000)	Males (*n* = 494)	Females (*n* = 506)	*P*‐value
Age (years)	50.4 (17.0)	50.2 (17.2)	50.6 (16.7)	0.743
Height (cm)	170.6 (9.7)	177.6 (7.1)	163.9 (6.6)	<0.001
Body mass (kg)	73.7 (14.8)	82.7 (12.6)	65.2 (11.2)	<0.001
BMI (kg/m^2^)	25.2 (3.8)	26.2 (3.3)	24.3 (3.9)	<0.001
Skeletal muscle index (kg/m^2^)	7.7 (1.5)	8.8 (1.1)	6.7 (1.0)	<0.001
Grip strength (kg)	37.6 (12.2)	46.5 (10.2)	29.1 (6.6)	<0.001
Plasma CAF concentration (pg/mL)	2603 (601.5)	2619.7 (696.1)	2587.6 (494.0)	0.404
Education, *n* (%)				
None or primary education	15 (1.5)	6 (1.2)	9 (1.8)	0.269
Lower secondary	58 (5.8)	34 (6.8)	24 (4.8)	
Higher secondary	164 (16.4)	85 (17.3)	78 (15.4)	
Third‐level degree	527 (52.7)	246 (49.7)	281 (55.5)	
Postgraduate degree	236 (23.6)	123 (24.9)	114 (22.5)	
No. of diseases/disorder, *n* (%)				
None	362 (36.2)	199 (40.2)	163 (32.3)	<0.001
One	276 (27.6)	138 (28.0)	138 (27.3)	
Two or more	362 (36.2)	157 (31.8)	205 (40.4)	
Habitual physical activity^a^	4.0 (2.2)	4.2 (2.3)	3.9 (2.2)	0.039
Alcohol consumption (units/week)	6.8 (6.5)	8.7 (7.5)	5.1 (4.8)	<0.001
Smoking status, *n* (%)				
Never smoked (<100 cigarettes)	590 (59.0)	271 (54.8)	319 (63.0)	0.014
Previously smoked (>100 cigarettes)	137 (13.7)	68 (13.8)	69 (13.7)	
Currently smoke (>100 cigarettes)	273 (27.3)	155 (31.3)	118 (23.4)	

BMI, body mass index; CAF, C‐terminal agrin fragment.

^a^ Days per week engaging in ≥30 min moderate intensity exercise.

### Reference values for plasma C‐terminal agrin fragment concentration across adulthood

**Figure 1:**
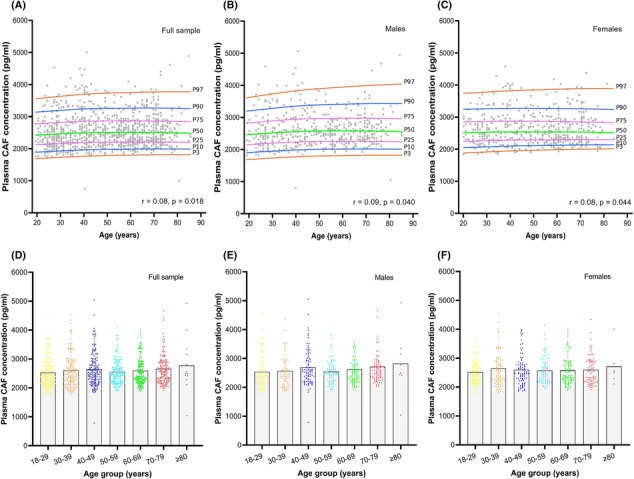
Percentile curves detailing the association between plasma C‐terminal agrin fragment (CAF) concentration and age (A–C); and plasma CAF concentrations according to 10‐year age‐groups throughout adulthood (D–F). Solid lines in (D)–(F) represent mean of each group; no statistically significant differences were observed.

Reference values for plasma CAF according to age and sex are detailed in Table 2, and corresponding percentile curves are illustrated in Figure 1. There were no significant differences in plasma CAF between males and females (*P* = 0.404). Overall, there was a significant, but modest increase in plasma CAF concentration with age (*P* = 0.018) (Figure 1). This positive association was observed for males (*P* = 0.040) and females (*P* = 0.044), and for both sexes, plasma CAF levels were lowest in the 18–29 year age group (males: 2539.6 pg/mL; females: 2518 pg/mL) and highest in the ≥80 year age group (males: 2825 pg/mL; females: 2712 pg/mL) (Figure 1). However, when stratified into 10‐year age categories, although differences in CAF concentration of up to 11.2% were observed (males: 18–29 years vs ≥ 80 years), none of the differences achieved statistical significance in either sex (Figure 1)

**Table 2:** Reference values for plasma C‐terminal agrin fragment (CAF) concentration across adulthood

Age	Sex	*n*	Plasma CAF concentrations (pg/mL)							
			Percentile							
			3rd	10th	25th	50th	75th	90th	97th	Mean (SD)
18–29	M	81	1852.5	1947.7	2151.0	2388.0	2811.3	3447.1	3717.5	2539.6 (570.8)
	F	78	1893.1	2069.1	2206.9	2436.8	2748.9	3190.3	3399.3	2518.9 (426.6)
30–39	M	79	1903.8	1976.0	2130.1	2461.9	2856.8	3376.2	3917.4	2569.6 (547.5)
	F	80	1868.1	2016.5	2232.7	2580.1	2918.0	3371.7	3986.3	2646.6 (583.3)
40–49	M	86	1931.7	2115.9	2222.1	2558.9	3006.6	3477.9	4011.5	2691.2 (635.9)
	F	81	1898.8	1989.3	2228.1	2479.3	2799.7	3362.8	3863.6	2600.7 (530.5)
50–59	M	78	1956.3	2098.2	2239.3	2479.4	2713.3	2993.5	3464.8	2541.6 (392.0)
	F	86	2021.5	2141.1	2229.9	2495.4	2823.9	3125.3	3570.9	2574.6 (440.5)
60–69	M	81	2004.8	2079.4	2236.8	2410.0	2772.7	3206.8	3447.3	2621.7 (980.0)
	F	94	1982.4	2094.9	2239.0	2400.6	2837.9	3365.2	3733.0	2578.3 (479.4)
70–79	M	81	2092.1	2163.4	2335.8	2565.6	2814.2	3255.1	4176.2	2719.4 (818.5)
	F	81	2004.4	2074.9	2190.8	2545.9	2893.0	3165.8	3564.6	2597.2 (483.2)
≥80^a^	M	8	‐	‐	‐	‐	‐	‐	‐	2825.0 (1097.9)
	F	6	‐	‐	‐	‐	‐	‐	‐	2712.6 (675.2)
Total	M	494	1921.0	2050.2	2229.0	2479.0	2837.3	3341.0	3832.0	2619.7 (696.1)
	F	506	1935.0	2052.7	2226.7	2479.3	2859.3	3313.4	3824.2	2587.6 (494.0)

^a^ Percentile values not shown for ≥80 age groups due to limited data.

### General associations between plasma C‐terminal agrin fragment concentration and skeletal muscle health in people aged 18–87 years

**Figure 2:**
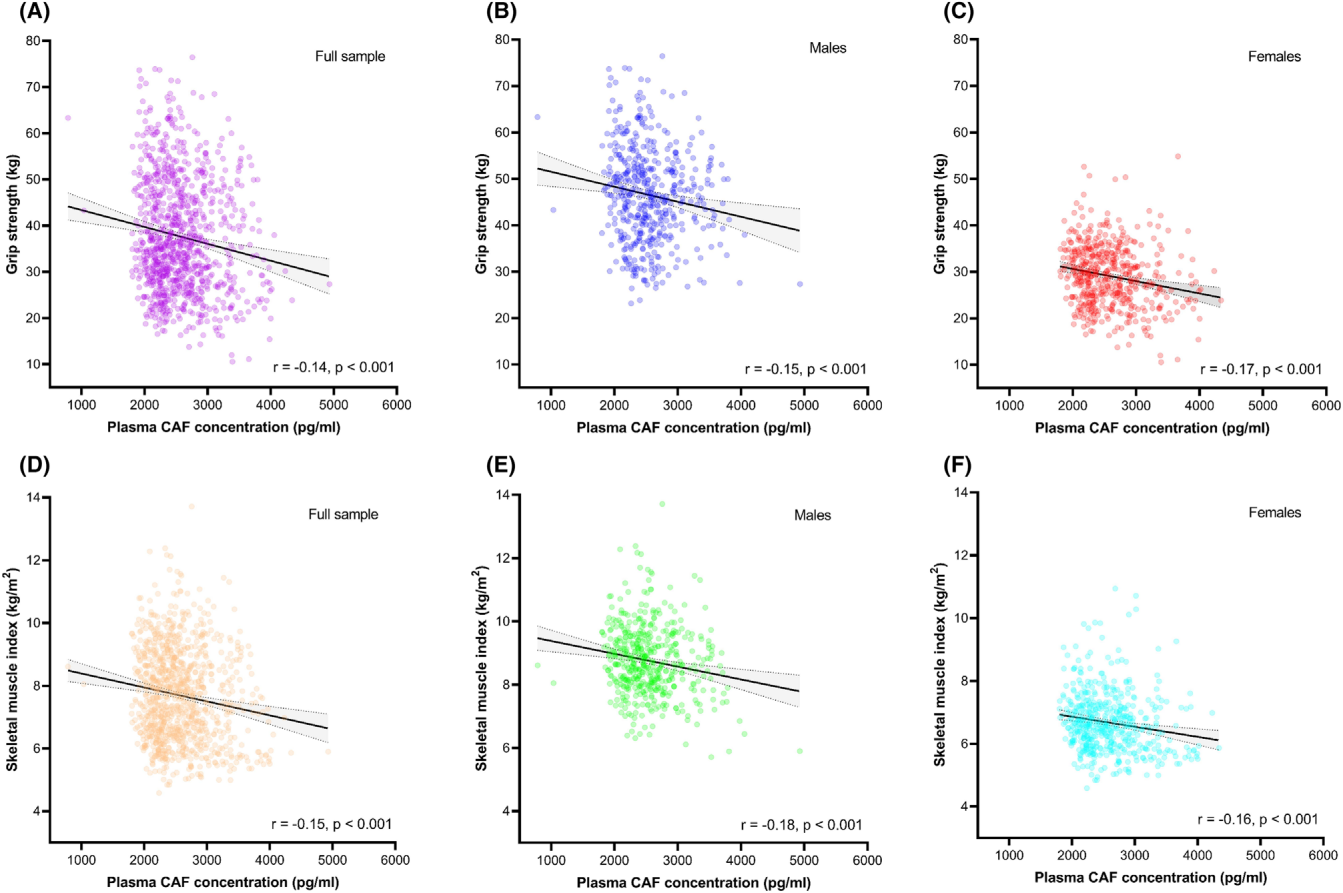
Correlations between plasma C‐terminal agrin fragment (CAF) concentration and grip strength (A–C) and skeletal muscle index (D–F). Grey shaded area depicts 95% confidence interval.

In the full sample, plasma CAF concentrations were negatively associated with grip strength (*r* = −0.14, *P* < 0.001) and SMI (*r* = −0.15, *P* < 0.001) (Figure 2). When stratified by sex, similar negative associations were observed in males (grip strength: *r* = −0.15, *P* < 0.001 and SMI: *r* = −0.18, *P* < 0.001), and in females (grip strength: *r* = −0.17, *P* < 0.001 and SMI: *r* = −0.16, *P* < 0.001). Similarly, linear regression revealed plasma CAF to be negatively associated with grip strength and SMI (both *P* < 0.001), when adjusted for sex and age (Table S2).

### Associations between plasma C‐terminal agrin fragment concentration, sarcopenia, and individual sarcopenia signatures in people aged ≥50 years

**Figure 3:**
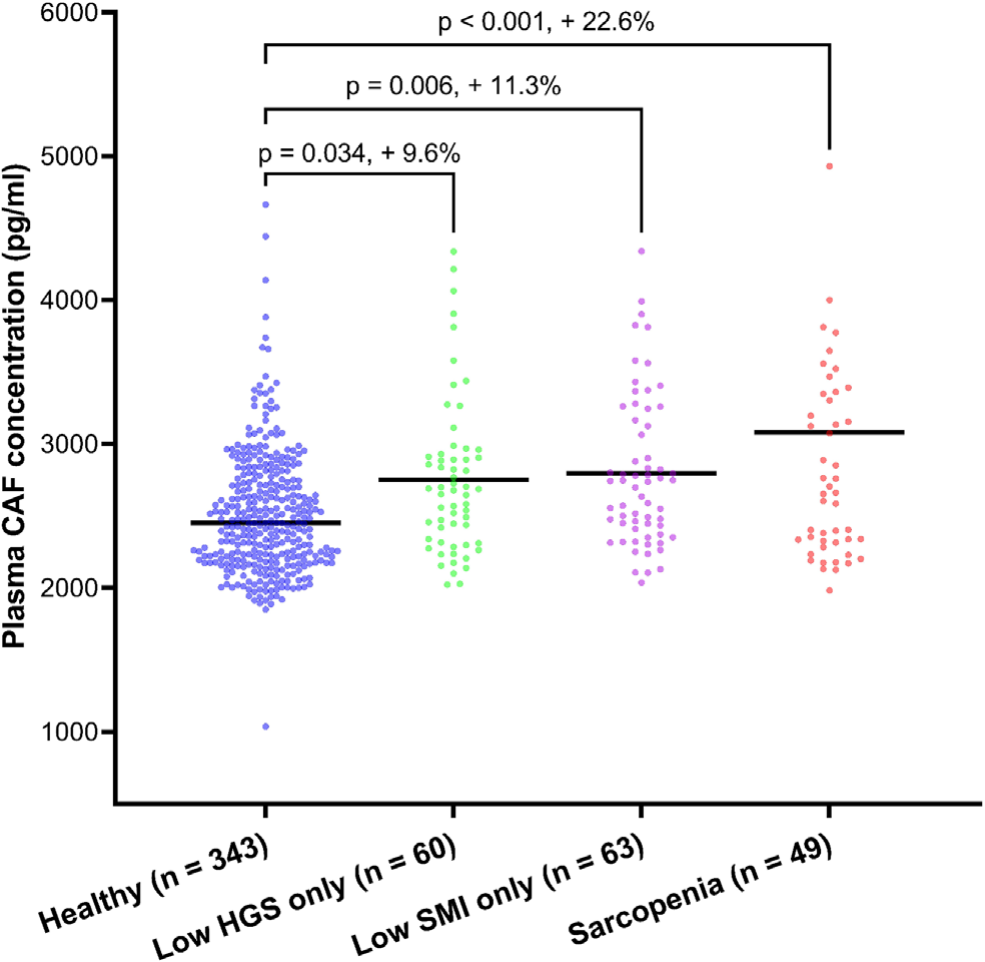
Difference in plasma C‐terminal agrin fragment (CAF) concentration according to sarcopenia status in people aged ≥ 50 years. HGS, hand grip strength; SMI, skeletal muscle index.

Out of a total of 515 people aged ≥50 years, 49 (9.5%) had sarcopenia, 63 (12.2%) had low SMI only, 60 (11.7%) had low grip strength only, and 343 (66.6%) were healthy. Plasma CAF concentrations were significantly higher in those with sarcopenia (+22.6%, *P* < 0.001), low SMI only (+11.3%, *P* = 0.006), and low grip strength only (+9.6%, *P* = 0.034), compared with healthy controls (Figure 3). These associations withstood adjustment for a panel of potential confounders including sex, age, BMI, habitual physical activity, educational attainment, alcohol consumption, smoking status, and co‐morbidity (*Table* *S3*).

When compared with those in the lowest plasma CAF concentration quartile (Q1), those in the highest quartile (Q4) had 3.25 greater odds for sarcopenia (95% CI = 1.41–7.49, *P* = 0.005), 2.76 greater odds for low SMI only (95% CI = 1.24–5.22, *P* = 0.012), and 2.56 greater odds for low grip strength only (95% CI = 1.07–5.57, *P* = 0.037) after controlling for several relevant confounders (*Table* *3*). When stratified according to CAF concentration *Z*‐score, those with a *Z*‐score ≥2 had 9.52 greater odds for sarcopenia (95% CI = 3.01–30.05, *P* < 0.001) while those with a *Z*‐score of 1–1.99 had 4.96 greater odds for sarcopenia (95% CI = 2.14–11.51, *P* < 0.001), compared with those with a *Z*‐score <1 (*Table* *S4*).

**Table 3:** Odds for sarcopenia and individual sarcopenia signatures according to plasma C‐terminal agrin fragment concentration quartile in people aged ≥50 years

Phenotype, *n* (%)	Quartile 1 (*n* = 129)	Quartile 2 (*n* = 129)	*P*‐value	Quartile 3 (*n* = 129)	*P*‐value	Quartile 4 (*n* = 128)	*P*‐value
Sarcopenia	8 (6.2)	12 (9.3)		9 (7.0)		20 (15.6)	
Model 1	1	1.32 (0.49–3.56)	0.502	0.89 (0.26–2.24)	0.879	3.16 (1.38–7.20)	0.006
Model 2	1	1.30 (0.47–3.51)	0.510	0.90 (0.25–2.73)	0.841	3.25 (1.41–7.49)	0.005
Low SMI only	6 (4.7)	14 (10.9)		16 (12.4)		24 (18.8)	
Model 1	1	1.38 (0.68–3.05)	0.467	1.59 (0.75–2.92)	0.231	2.74 (1.19–5.14)	0.019
Model 2	1	1.33 (0.65–2.93)	0.470	1.56 (0.79–2.98)	0.247	2.76 (1.24–5.22)	0.012
Low HGS only	8 (6.2)	13 (10.1)		17 (13.2)		22 (17.2)	
Model 1	1	1.65 (0.64–3.09)	0.233	2.02 (0.84–3.39)	0.128	2.64 (1.10–5.71)	0.029
Model 2	1	1.62 (0.62–2.97)	0.237	2.06 (0.85–3.43)	0.114	2.56 (1.07–5.57)	0.037

Quartile 1 = reference; Model 1 = adjusted for sex, age, body mass index; Model 2 = model 1 plus smoking status, alcohol consumption, educational attainment, activity level, co‐morbidity. Quartile ranges were age‐ and sex‐specific; refer to Table 2.

HGS, handgrip strength; SMI, skeletal muscle index.

**Figure 4:**
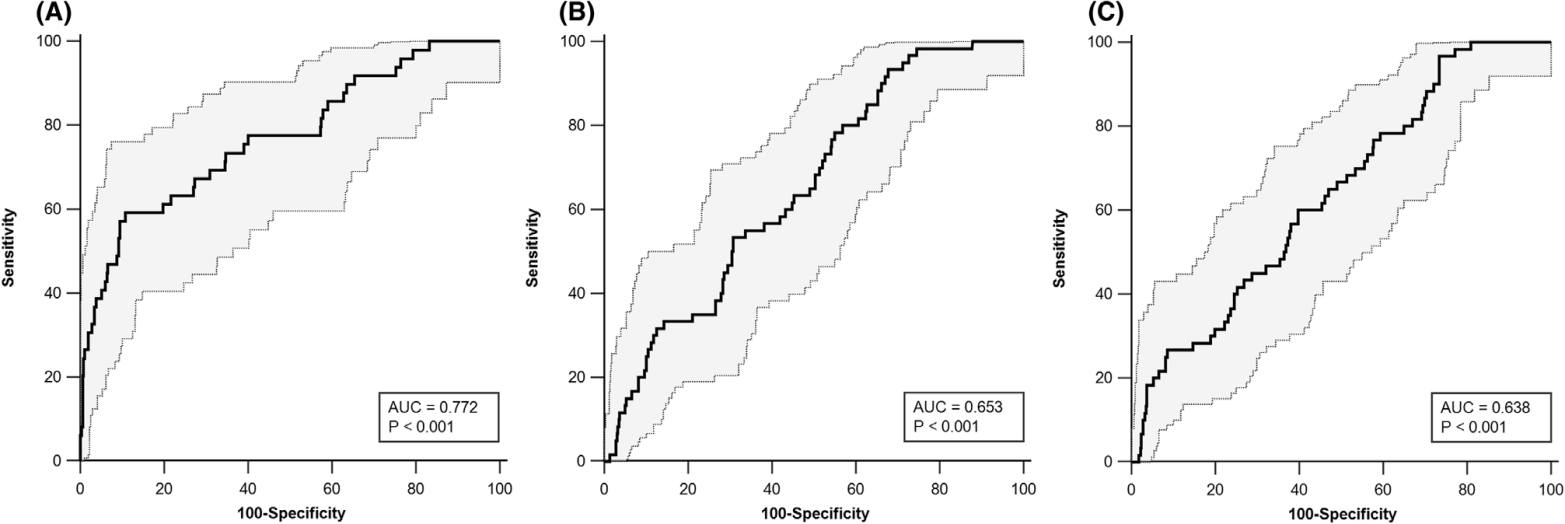
Receiver operating characteristic analysis of C‐terminal agrin fragment for sarcopenia (A), low skeletal muscle index only (B), and low grip strength only (C). Grey shaded area depicts 95% confidence interval.

Receiver operator characteristic analysis revealed plasma CAF concentration to have acceptable diagnostic accuracy for sarcopenia with an area under the curve (AUC) of 0.772 (95% CI = 0.733–0.807, *P* < 0.001), and optimal cut‐point of 3073.1 pg/mL (59.2% sensitivity and 89.3% specificity) (Figure 4). The AUCs for low SMI and low grip strength were 0.653 (95% CI = 0.610–0.694, *P* < 0.001) and 0.638 (95% CI = 0.595–0.680, *P* < 0.001), with optimal cut‐points of 2734.6 pg/mL (53.4% sensitivity and 69.2% specificity) and 2645.0 pg/mL (60.0% sensitivity and 60.2% specificity).

## Discussion

Increasing interest surrounds the potential utility of blood biomarkers for enhancing strategies to reduce the burden of sarcopenia. Existing data suggest that CAF is amongst the most promising candidates, although a lack of reference data has thus far impeded a more widespread incorporation of its assessment in healthy adults. Herein, we present detailed sex‐ and age‐specific reference values for plasma CAF concentration from a large (*n* = 1000), well‐characterized cohort of healthy adults aged from 18 to 87 years. These data, which may guide the interpretation of plasma CAF concentrations across adulthood, represent the largest sample of circulating CAF values reported to date.

A modest, yet significant positives association was observed between plasma CAF concentration and age in both sexes, with the lowest values being observed in 18‐ to 29‐year‐old people and the highest values in people ≥80 years old. These findings are consistent with existing reports of a gradual, age‐related increase in circulating CAF level in healthy adults,^15^, ^22^ reflecting both the relative stability of the NMJ throughout much of the human lifespan, and the direct relationship between circulating CAF and NMJ integrity.^23^, ^24^ Nevertheless, although plasma CAF concentrations do not appear to be robustly linked with age, differences of up to approximately 11% between certain age groups suggest that the detailed normative data presented in this study may be useful for interpreting concentrations at different stages throughout adulthood.

Unlike its association with age, circulating CAF level is strongly linked with skeletal muscle health, with higher concentrations indicating poorer skeletal muscle health.^13^, ^15^ This phenomenon is a physiological by‐product of the robust relationship between NMJ integrity and skeletal muscle health. It is well‐established that the NMJ serves an indispensable role in regulating skeletal muscle, and so a decline in NMJ integrity and consequent deterioration in skeletal muscle status manifests itself in the bloodstream as enhanced CAF concentrations.^13^, ^24^ The present study provides supporting evidence for this, by highlighting strong associations between plasma CAF level and skeletal muscle phenotypes across adulthood, and specifically in people ≥50 years who are particularly vulnerable to sarcopenia. Across adulthood, we found plasma CAF concentrations to be negatively associated with grip strength and SMI, represented by significant, but relatively modest correlation coefficients (*r* = −0.14 to *r* = −0.18). Although a paucity of data of circulating CAF levels across adulthood impedes the contextualization of our data, similar correlations have been observed in older adults.^11^ With this in mind, the association between circulating CAF and skeletal muscle health appears to be relatively linear and modest throughout adulthood, when skeletal muscle phenotypes are considered as continuous variables.

Considerably stronger associations are apparent when skeletal muscle health is categorized according to specific thresholds of degradation, such as those used for sarcopenia diagnosis.^19^ Indeed, amongst the most noteworthy findings of this study are the robust associations between circulating CAF concentration and sarcopenia in middle‐aged and older adults. Specifically, plasma CAF levels were 22.6% higher in people with sarcopenia and up to 11.3% higher in people presenting with individual sarcopenia signatures (low SMI only or low grip strength only), compared with healthy controls. These findings showcase a robust progressive relationship between skeletal muscle degradation and circulating CAF. Although existing research broadly favours a strong connection between circulating CAF and skeletal muscle health, some inconsistent findings have been observed (see Monti et al.^10^). However, important covariates such as habitual physical activity and/or co‐morbidities have often been uncontrolled for in existing studies, which may influence the accuracy of reported associations.^10^ Our study adds considerable weight to existing data, by presenting results from a comprehensively characterized cohort some three‐fold larger than any existing study, demonstrating a strong relationship between plasma CAF concentration and skeletal muscle integrity in healthy older adults. Furthermore, we demonstrate that the reference data presented herein may have clinical value for identifying people with poor skeletal muscle health, who may benefit from therapeutic interventions. Indeed, when compared with the lowest quartile, people with CAF concentrations in the highest quartile had 3.25 higher odds for sarcopenia, 2.76 higher odds for low SMI only, and 2.56 higher odds for low grip strength only. Stratification by *Z*‐score revealed further encouraging findings, with a *Z*‐score of ≥2 and 1–1.99 carrying 9.52 greater odds and 4.96 greater odds for sarcopenia, compared with a *Z*‐score <1. It is worth noting, however, that the sample sizes of these *Z*‐score groups were small and so, the results should be interpreted in light of that. Furthermore, plasma CAF concentration only displayed acceptable diagnostic accuracy for sarcopenia, and not individual sarcopenia components. Additional research is needed, therefore, to further illuminate the diagnostic potential of plasma CAF for different categories of skeletal muscle health. Despite such areas warranting future research, our findings, in combination with existing data, clearly demonstrate a strong connection between circulating plasma CAF and skeletal muscle status.

The relevance of assessing CAF concentrations is further supported by data demonstrating the sensitivity of circulating CAF to acute periods of disuse and consequent skeletal muscle degradation. For example, notable increases of 5.4% in circulating CAF concentration have been observed in humans following 10 days unilateral lower limb suspension (ULLS)^25^ and of 19.4% following 10 days bedrest.^26^ These findings show circulating CAF to be a sensitive marker of disuse‐induced NMJ deterioration, responsive to the severity of disuse (greater rise in circulating levels following bedrest vs ULLS), and correlated with decrements in skeletal muscle morphology and function. As such, circulating CAF assessment may present additional value in screening the neuromuscular health of people during periods of disuse such as bedrest, which may become particularly relevant in older adulthood.

Although notable variation in reported blood CAF concentrations permeates existing literature, much of this is explained by studies including participants suffering, for example, from conditions such as hip fracture,^27^ acute and chronic heart failure,^28^, ^29^ kidney disease,^22^ and chronic obstructive pulmonary disease^30^ with significant variation in CAF concentrations. In contrast, studies focussing on ‘healthy’ adults have observed more consistent values, similar to those reported in the present study (ranging between 1700 and 5000 pg/mL; summarized in Monti et al.^10^). Although even this range appears somewhat large upon initial consideration, it is important to note that due to the relationship between circulating CAF and skeletal muscle health, most existing studies relate specifically to older adults, and many of these have included subjects at a more advanced stage of musculoskeletal decline than in the present study, which may somewhat help to explain the variance in CAF concentrations. As such, we believe the reference values presented herein to be suitable for interpreting plasma CAF concentrations in healthy community‐dwelling men and women across adulthood. Nevertheless, we recognize that more studies establishing reference values for circulating CAF concentrations in other populations, derived from large age‐diverse cohorts, are urgently needed to contextualize our findings. Establishing reference data for healthy and diseased populations will ultimately allow for a more widespread assessment of CAF in our society.

Several strengths and limitations of our work should be acknowledged. The strengths include (1) the large sample size, equally representative of males and females, spanning a wide age‐range across adulthood (18–87 years); (2) comprehensive cohort phenotyping, allowing for statistical analyses to be appropriately adjusted for a range of covariates, several of which have not been accounted for in previous studies. The limitations include (1) normative data were established cross‐sectionally, and so the percentile curves should only be used as a guide for the potential trajectory of plasma CAF concentrations across adulthood; (2) the cross‐sectional design also prevents causality between plasma CAF and skeletal muscle traits to be established; (3) DXA is inherently prone to underestimating age‐related loss of skeletal muscle mass, unlike magnetic resonance imaging that may have provided a more accurate measure of SMI^31^; (4) our findings are based upon people residing in Ireland, and so the transferability of these data to other populations remains to be elucidated; (5) future studies may benefit from examining the potential mediating effect of pharmacological therapy on the association between plasma CAF concentrations and skeletal muscle health.

In conclusion, we have generated detailed reference values for circulating CAF concentration across adulthood that may help identify people who are vulnerable to poor skeletal muscle outcomes for inclusion in primary care interventions. Our study adds clarity to existing data, by presenting evidence of a strong relationship between circulating CAF concentrations and signatures of sarcopenia within a large, well‐characterized cohort of adults. Collectively, our findings support the use of circulating CAF concentration as a marker of skeletal muscle health, particularly in the context of sarcopenia. The normative data detailed herein provide a strong foundation for further exploration into the clinical utility of plasma CAF, and it is our hope that this study stimulates the establishment of reference data from other populations that are ultimately needed for a more widespread incorporation of circulating CAF assessment into routine medical practice. Doing so may enhance existing strategies seeking to promote skeletal muscle health across the lifespan, by providing an alternative, cost‐effective and accessible screening method.

## Conflict of interest

All authors declare no conflicts of interest.

## Funding

We acknowledge co‐funding from Next Generation EU (DM 1557 11.10.2022) to MVN, in the context of the National Recovery and Resilience Plan, Investment PE8 – Project Age‐It: ‘Ageing Well in an Ageing Society’. The views and opinions expressed are only those of the authors and do not necessarily reflect those of the European Union or the European Commission. Neither the European Union nor the European Commission can be held responsible for them. All authors certify that they comply with the ethical guidelines for authorship and publishing in the *Journal of Cachexia, Sarcopenia and Muscle*.

## Supporting information


**Table S1.** Differences in plasma C‐terminal agrin fragment (CAF) concentration, grip strength and skeletal muscle index according to disease/disorder prevalence, stratified by 10‐year age groups
**Table S2.** Association between plasma C‐terminal agrin fragment (CAF), grip strength and skeletal muscle index (SMI) in people aged 18–87 years
**Table S3.** Adjusted associations between plasma C‐terminal agrin fragment (CAF) concentration and sarcopenia status in people aged ≥ 50 years
**Table S4.** Odds for sarcopenia according to plasma C‐terminal agrin fragment concentration Z‐score in people aged ≥ 50 years
