# Supplementary material for: Plasma C‐terminal agrin fragment concentrations across adulthood: Reference values and associations with skeletal muscle health
Source: J Cachexia Sarcopenia Muscle. 2024 Jun 7;15(4):1501–10. doi: 10.1002/jcsm.13507 (PMC11294022; doi:10.1002/jcsm.13507)
Supplement: Supplementary file 1 — Table S1. Differences in plasma C‐terminal agrin fragment (CAF) concentration, grip strength and skeletal muscle index according to disease/disorder prevalence, stratified by 10‐year age groups Table S2. Association between plasma C‐terminal agrin fragment (CAF), grip strength and skeletal muscle index (SMI) in people aged 18–87 years Table S3. Adjusted associations between plasma C‐terminal agrin fragment (CAF) concentration and sarcopenia status in people aged ≥ 50 years Table S4. Odds for sarcopenia according to plasma C‐terminal agrin fragment concentration Z‐score in people aged ≥ 50 years [file JCSM-15-1501-s001.docx]

**List of diseases and disorders screened**

*The number of diseases and/or disorders present was recorded for each participant.*

- **Cancer**: blood leukaemia, breast, colon, kidney, lung, ovarian, pancreas, prostate, skin
- **Heart disease/disorder:** abnormal heart beat arrhythmia, angina, heart attack, high blood pressure, high cholesterol, stroke
- **Skin disorder**: eczema, melasma, psoriasis, rosacea, scleroderma
- **Digestive/bowel disorder**: coeliac disease, Crohn’s disease, fatty liver disease, inflammatory bowel disease, irritable bowel syndrome, ulcerative colitis
- **Breathing disorder**: asthma, chronic obstructive pulmonary disease, emphysema, cystic fibrosis, idiopathic pulmonary fibrosis, lung fibrosis, sarcoidosis
- **Bone/joint disorder**: ankylosing spondylitis spondylarthritis, enteropathic arthritis, osteoarthritis, psoriatic arthritis, reactive arthritis, rheumatoid arthritis, osteoporosis
- **Pain disorder**: chronic back pain, migraine
- **Mental health condition**: anxiety, bipolar disorder, depression, schizophrenia
- **Neurological**: Alzheimer’s, age related macular degeneration, autism, epilepsy, motor neurone disease, multiple sclerosis, Parkinson’s
- **Diabetes**: gestational diabetes, type 1 diabetes, type 2 diabetes
- **Other**: alopecia, endometriosis, haemochromatosis, kidney disease, kidney failure, Lupus, Sjogren Syndrome, thyroid disorder, other (please specify)

| **Supplementary Table 1. Differences in plasma C-terminal agrin fragment (CAF) concentration, grip strength and skeletal muscle index according to disease/disorder prevalence, stratified by 10-year age groups** | | | | |
| --- | --- | --- | --- | --- |
| **Age group (years)** | **Number of diseases/disorders** | | | |
|  | 0 | 1 | 2 or more | p-value |
|  | *Plasma CAF (pg/ml)* |  |  |  |
| 18-29 | 2539.6 (54.5) | 2453.3 (76.7) | 2612.0 (93.4) | 0.409 |
| 30-39 | 2618.1 (67.3) | 2490.8 (86.9) | 2721.4 (89.1) | 0.181 |
| 40-49 | 2591.7 (71.9) | 2710.5 (86.8) | 2663.4 (80.9) | 0.559 |
| 50-59 | 2509.4 (57.3) | 2596.5 (59.6) | 2571.1 (56.7) | 0.553 |
| 60-69 | 2540.1 (112.5) | 2547.0 (101.4) | 2645.2 (79.1) | 0.651 |
| 70-79 | 2611.8 (128.3) | 2725.4 (131.7) | 2775.7 (86.4) | 0.571 |
| ≥80 | 2319.3 (464.7) | 3102.3 (536.6) | 2898.8 (351.3) | 0.504 |
|  | *Grip strength (kg)* |  |  |  |
| 18-29 | 41.89 (1.34) | 38.45 (1.92) | 43.71 (2.31) | 0.177 |
| 30-39 | 42.71 (1.40) | 38.81 (1.81) | 37.63 (1.85) | 0.061 |
| 40-49 | 45.1 (1.48) | 41.61 (1.79) | 38.26 (1.67) | 0.010 |
| 50-59 | 39.69 (1.65) | 38.66 (1.72) | 35.87 (1.64) | 0.240 |
| 60-69 | 34.39 (1.76) | 34.97 (1.59) | 33.39 (1.24) | 0.722 |
| 70-79 | 31.65 (1.51) | 33.58 (1.55) | 29.05 (1.02) | 0.042 |
| ≥80 | 34.48 (3.43) | 24.35 (3.96) | 26.91 (2.59) | 0.151 |
|  | *Skeletal muscle index (kg/m^2^)* | | |  |
| 18-29 | 8.13 (0.18) | 7.93 (0.25) | 8.26 (0.30) | 0.663 |
| 30-39 | 8.19 (0.18) | 7.89 (0.23) | 7.44 (0.23) | 0.041 |
| 40-49 | 8.18 (0.19) | 7.92 (0.23) | 7.64 (0.21) | 0.170 |
| 50-59 | 7.85 (0.19) | 7.65 (0.20) | 7.50 (0.19) | 0.436 |
| 60-69 | 7.33 (0.22) | 7.57 (0.20) | 7.24 (0.15) | 0.407 |
| 70-79 | 7.24 (0.19) | 7.56 (0.20) | 6.99 (0.13) | 0.057 |
| ≥80 | 7.49 (0.39) | 6.62 (0.45) | 6.82 (0.29 | 0.297 |
| Data presented as mean (standard error of mean) | | | | |

| **Supplementary Table 2. Association between plasma C-terminal agrin fragment (CAF), grip strength and skeletal muscle index (SMI) in people aged 18-87 years** | | | | |
| --- | --- | --- | --- | --- |
| **Dependent variable ^a^** | *Plasma CAF (pg/ml)* | | | |
|  | β | 95% CI | p-value | R^2^ |
| Grip strength | -0.001 | -0.002 - -0.001 | <0.001 | 0.607 |
| SMI | -0.001 | -0.001 - -0.001 | <0.001 | 0.550 |
| ^a^ = adjusted for sex and age | | | | |

| **Supplementary Table 3. Adjusted associations between plasma C-terminal agrin fragment (CAF) concentration and sarcopenia status in people aged ≥ 50 years** | | | | | |
| --- | --- | --- | --- | --- | --- |
| **Model** | | **Healthy (n=343)** | **Sarcopenia (n=49)** | **Low SMI (n=63)** | **Low HGS (n=60)** |
|  | | *Plasma CAF (pg/ml)* | | | |
| Model 1 | 2522.7 (36.3) | | 3115.1 (102.4)*** | 2781.9 (56.5)*** | 2710.2 (63.5)* |
| Model 2 | 2529.8 (36.4) | | 3094.4 (103.2)*** | 2776.8 (57.1)*** | 2704.1 (64.6)* |
| Data presented as mean (standard error of mean); Model 1 = adjusted for age, sex and body mass index; Model 2 = Model 1 plus physical activity, alcohol consumption, smoking status, educational attainment and comorbidity; SMI = skeletal muscle index; HGS = handgrip strength * p<0.05; *** p<0.001 | | | | | |

| **Supplementary Table 4. Odds for sarcopenia according to plasma C-terminal agrin fragment concentration Z-score in people aged ≥ 50 years** | | | | | |
| --- | --- | --- | --- | --- | --- |
| **Model** | **Z-score** |  |  |  |  |
|  | **<1 (n=451)** | **1-1.99 (n=44)** | **p-value** | **≥ 2 (n=20)** | **p-value** |
| *Sarcopenia, n (%)* | 32 (7.1) | 11 (25.0) |  | 6 (30.0) |  |
| Model 1 | 1 | 4.96 (2.14-11.51) | <0.001 | 9.52 (3.01-30.05) | <0.001 |
| Z-score <1 = reference; Model 1 = adjusted for sex, age, body mass index | | | | | |
